# To Perceive or Not Perceive: The Role of Gamma-band Activity in Signaling Object Percepts

**DOI:** 10.1371/journal.pone.0066363

**Published:** 2013-06-13

**Authors:** João Castelhano, José Rebola, Bruno Leitão, Eugenio Rodriguez, Miguel Castelo-Branco

**Affiliations:** 1 Visual Neuroscience Laboratory, Institute for Biomedical Imaging and Life Sciences, Faculty of Medicine, University of Coimbra, Coimbra, Portugal; 2 Center for Informatics and Systems, University of Coimbra, Coimbra, Portugal; 3 Max-Planck for Brain Research, Frankfurt am Main, Germany; and Pontificia Universidad Católica de Chile, Escuela de psicología, Santiago, Chile; University of British Columbia, Canada

## Abstract

The relation of gamma-band synchrony to holistic perception in which concerns the effects of sensory processing, high level perceptual gestalt formation, motor planning and response is still controversial. To provide a more direct link to emergent perceptual states we have used holistic EEG/ERP paradigms where the moment of perceptual “discovery” of a global pattern was variable. Using a rapid visual presentation of short-lived Mooney objects we found an increase of gamma-band activity locked to perceptual events. Additional experiments using dynamic Mooney stimuli showed that gamma activity increases well before the report of an emergent holistic percept. To confirm these findings in a data driven manner we have further used a support vector machine classification approach to distinguish between perceptual *vs.* non perceptual states, based on time-frequency features. Sensitivity, specificity and accuracy were all above 95%. Modulations in the 30–75 Hz range were larger for perception states. Interestingly, phase synchrony was larger for perception states for high frequency bands. By focusing on global gestalt mechanisms instead of local processing we conclude that gamma-band activity and synchrony provide a signature of holistic perceptual states of variable onset, which are separable from sensory and motor processing.

## Introduction

Oscillatory processes in the gamma frequency range have been proposed to play a role in percept formation and object representation. Studies using EEG and MEG [1]–[3] have suggested that gamma-band oscillations are related to integration of information and the ability to form coherent gestalts [1], [2] as well as attention and working memory processes [3]–[10]. Gamma-band synchrony does indeed seem to reflect binding of information across different brain regions [11]–[16] that leads to the emergence of a coherent percept [17]–[19]. Studies of oscillatory patterning may be important to understand normal and abnormal cognitive function related to perceptual functions [20]. Although some studies reported that gamma-activity may be influenced by artifacts of muscle activity and eye movements [21]–[23], valuable methods to attenuate this problem have been developed [24]–[26].

Here we examined the oscillatory large-scale neural correlates of gestalt-like perceptual recognition moments. As previous studies have relied on simple contrasts across inverted *vs.* upright static stimuli [17] we aimed to study the moment a coherent visual percept is formed. Paradigms where object recognition is variable in time would be helpful in elucidating this issue by isolating a neural correlate of coherent perception. Neurochronometric paradigms [27] that allow the emergence of variable moments of perception would also help in further clarifying the role of gamma-band synchrony in gestalt based perception.

To help identify the processes underlying the emergence of a coherent object/face percept under ambiguous stimulus conditions, and the role of gamma-band activity in these processes, we have recorded EEG/ERP signals while performing two different tasks that used Mooney objects (two-tone black and white degraded images, [28]). The first task tried to achieve that goal with rapid serial visual presentation paradigms, where short lived target Mooney stimuli do appear randomly with low (1/30) probability. In a second experiment, based on time variable percepts, we designed a new face paradigm [29] that takes advantage of the well known role of face inversion in holistic processing [27], [30]–[32]. A configural/holistic-based processing mode operates for upright faces [33] and a part-based processing mechanism is activated when faces are inverted [27], [34]. We used such a paradigm that delayed the time of recognition from stimulus onset [29]. We were able to observe a delayed transition from non perception to perceptual states reflecting a gradual transition from local to holistic processing. This leads to the simple prediction that gamma should increase during the transition moment.

We aimed to investigate the link between gamma-band activity and the aforementioned moment whereby a coherent visual percept is formed, which helped dissociating low level visual analysis from high level categorical perception. We used the time-frequency and phase synchrony analysis described elsewhere [17], [35]–[37]. Finally, we performed an additional independent validation by a data driven (non hypothesis constrained) approach using support vector machine (SVM) classification tools [38]. We were able to differentiate between perceptual states based on temporal activity patterns and thereby support their likely functional relation with object recognition.

Given that previous studies have shown that the brain codes different information in multiple oscillatory bands [39]–[41], we did analyze a broad range of frequency bands, beyond the usual focus on high-frequency (60–75 Hz) as well as lower frequency (30–45 Hz) EEG low gamma bands and high beta [42]. We tested whether induced (non-phase locked) oscillatory activity might be differently modulated depending on the particular frequency band as a function of perceptual state, as tested using statistical classification tools.

## Materials and Methods

### Ethics Statement

**Figure 1:**
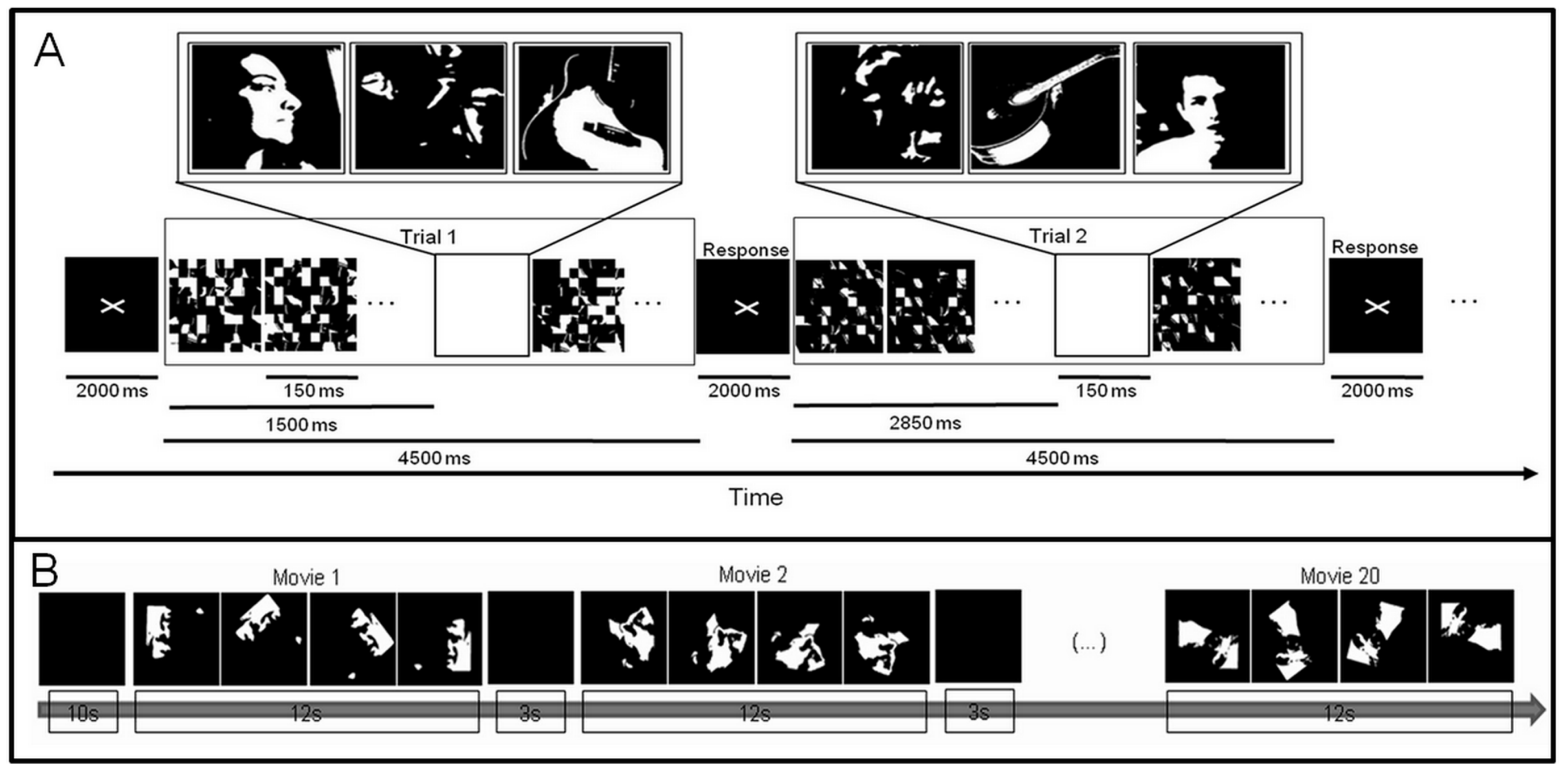
Summary of the tasks. A: EEG Mooney rapid visual presentation task, with delayed response. Meaningful (perceived as faces or guitars) objects appear among noise images. Mooney faces and Mooney guitars are shown randomly with a likelihood of 1/30 (between the 10^th^ and 20^th^ presented images) at each trial masked backward and forward by a randomization of itself (each picture 150 ms). Subjects had to report the presence of a target (Mooney face or Mooney Guitar or none of these objects) at the end of the trial (inter-stimulus interval is 2000 ms). B: Mooney dynamic stimuli - Time-line of one run; for clarity, representative snapshots are represented in separated boxes (in the experiment movies run continuously and smoothly). Accordingly, only 4 snapshots are shown for each movie – faces rotate from inverted to upright in 12 s movies separated by a 3 s black screen. Subjects were instructed to provide a motor report, when they perceived the face, as quickly as possible.

This study and all the procedures were reviewed and approved by the Ethics Commission of the Faculty of Medicine of the University of Coimbra (Comissão de Ética da Faculdade de Medicina da Universidade de Coimbra) and was conducted in accordance with the declaration of Helsinki. Written informed consent was obtained from all participants. The subjects of the photographs in figure 1 have given written informed consent, as outlined in the PLOS consent form, for publication of their photograph.

### Participants

All participants (*n* = 20, mean age 27.78±4.88 years, 9 males and 11 females; 2 left-handed) had normal or corrected-to normal vision and no history of neurological disorders. All participants were naive regarding the purpose of the study. Participants took part in EEG recordings, distributed over 2 experiments (14 in experiment 2, 8 in experiment 1). Two of these subjects underwent both experiments.

### Experimental paradigms

#### Experiment 1– Perceptual reports on briefly presented ambiguous stimuli – perceptual task with delayed response

EEG/ERP data were recorded along with a rapid visual presentation task with target probability of 1/30 (see below). Subjects (n = 8) performed a three way forced choice task where they had to report the presence of a target (Mooney face or Mooney Guitar or none of these objects) among frequent stimuli at the end of the trial (delayed report moment; see Figure 1A). The choice of guitars was due to the fact that they have a highly prototypical shape, like faces. Using these stimuli it is possible to modify local features without changing global configuration. Moreover there is little correspondence between face-selective areas and regions correlated with guitar identification [43]. Frequent stimuli were scramble versions of the target. Each picture was presented briefly for 150 ms in trials of 4500 ms. Each trial contained a target and 29 random versions of it (frequent standard stimuli). 103 different target Mooney faces and 103 target Mooney guitars were shown (subjects did not know when the target stimulus would appear). The timing of target presentation was variable between 1500 ms and 3000 ms in the trial. Subjects performed 7 runs (6 runs with 30 trials and 1 run with 26 trials) with trials randomized across subjects.

Stimuli were generated in Psychophysics Toolbox (running in Matlab®) to enable calculation on the fly of different random pictures (standard distractors – frequent stimuli). Stimuli were presented in a black background in a CRT monitor with a resolution of 1024×768 pixels and refresh rate of 85 Hz. They spanned a visual angle of 4.5°×4.5°.

#### EEG Recording– experiment 1

Subjects sat in a comfortable chair in a darkened room at a viewing distance of 120 cm from the stimulus presentation monitor.

We have used a 128 channel EEG system (Compumedics Quick cap; NeuroScan, USA) for recording. Caps were interfaced through SynAmps2 (NeuroScan, USA) signal amplifier, which fed the signal through the Acquire Data Acquisition software (version 4.3.1, Compumedics NeuroScan, USA) at a sampling rate (SR) of 2000 Hz. No notch filters were used during recording and impedances were kept under 10 kΩ (electrodes with higher impedances were marked as bad). All electrodes were referenced during recording to one reference electrode located close of CZ. Data were stored in the Portuguese Brain Imaging Network repository.

#### EEG analysis– experiment 1

We used Edit EEG/ERP analysis software (version 4.5, NeuroScan, USA) for data pre-processing and extraction of event related responses (target stimulus locked event related potentials (ERP)). The channels that did not fulfill the impedance criteria during acquisition were rejected. Offline re-referencing, using average reference was then performed [35]. Data were digitally high-pass filtered at 1 Hz using a finite impulse response filter and amplitude based (−75, 75 µV) artifact rejection routines were then applied. Blinks were removed by rejecting epochs in which the electrooculogram bipolar channels exceeded ±100 µV. Stimulus presentation time (150 ms) was below the known latency of eye movements. Miniature saccades usually show an average peak around 250 ms [44]. Data were filtered with a low-pass filter of 100 Hz and then segmented into epochs (−200 ms to 800 ms) locked to the onset of the target stimuli (Mooney faces and guitars). Only trials with correct categorization of the stimuli (>90%) were considered for analysis. We used Matlab®(v.R2010a, The MathWorks, USA) and EEGLAB Matlab® toolbox (version 10.2.5.6b) for time-frequency, phase synchrony and additional statistical analysis (see below for details).

We performed source localization for the grand average ERPs of the target stimuli. Source analysis was performed in Curry 5.0 software (NeuroScan, USA) on a realistic head model. Group average ERP data was co-registered with anatomical magnetic resonance (MR) data using landmarks and applying standard xyz coordinates of the channel locations. A boundary element model (BEM) was created from standard anatomical MR data and current source density was estimated for the ERP peaks with no assumption regarding the number or location of active sources. The sLORETA algorithm (standardized low resolution brain electromagnetic tomography) was used [45], [46]. This method is a standardized discrete, three-dimensional (3D) distributed, linear, minimum norm inverse solution. It takes several neurophysiologic and anatomical constraints into account and has been shown to yield images of standardized current density with exact localization in the presence of measurement and biological noise [46].

#### Experiment 2– Perceptual reports on dynamic ambiguous two-tone (Mooney) stimuli - emergent percepts and concomitant responses

In this experiment, using dynamic stimuli, EEG data were recorded in 14 right-handed subjects. Rotating Mooney face movies – starting from inverted to upright – were presented. The starting inverted position ensured minimal likelihood of initial face perception and induced late recognition in a substantial proportion of trials (see behavioural results). This is a new paradigm (see Rebola et al., 2012 [29]) where the 2-tone images started in the inverted position (180°), where local processing prevails, and slowly rotate for 9 s until they reach the upright position (0°), favoring holistic processing, staying then fixed for 3 s. This design enabled the presence of different perceptual states for the same physical stimulus (starting from absent perception). This way one can compare between distinct perceptual states induced by the same stimulus and not only a simple contrast between different pictures. Each movie contained a single embedded face and every stimulus appeared only once during the experiments to prevent repetition effects (for data on detection rates see below). We choose only one category (faces) because the initial inverted configuration has only been proven to be non-holistic for faces [27], [47]. For this stimulus category there is a transition from nonholistic/non perception to holistic/perceptual states. Stimuli were presented with Presentation (version 12.1, Neurobehavioral Systems, Albany, CA, USA) in a setup similar to experiment 1. The experiment was divided in 5 runs (total of 103 trials; 4 runs with 20 trials each and the last run with 23 trials). All runs started with a fixation period of 10 s followed by a Mooney dynamic face stimulus which was presented for 12 seconds. Stimuli were separated (inter stimulus interval) by a black screen during 3 s. Figure 1B summarizes the experimental paradigm. Subjects were instructed to search for a face and to press a button (concomitant report moment) as quickly as possible, only when they were confident of its presence. Stimuli remained visible until the end (12 seconds) for the perceived trials (even after the response) and for the trials that never came to a full percept.

#### EEG Recording- experiment 2

Subjects sat in a comfortable reclining chair in a darkened, acoustically and electrically shielded room at a viewing distance of 120 cm from the stimulus presentation monitor.

We have used a 64 channel system (Compumedics Quick cap; NeuroScan, USA) for recording. Electrodes were displayed as the 10–20 system in caps that were interfaced through SynAmps2 signal amplifier, which fed the signal through the Acquire Data Acquisition software (version 4.3.1, Compumedics NeuroScan, USA) at a SR of 2000 Hz. There were no filters applied during recording and impedances were kept under 10 kΩ (electrodes with higher impedances were marked as bad). Reference was set to one reference electrode located close of CZ.

#### EEG analysis- experiment 2

We used Edit EEG/ERP analysis software (version 4.5, Compumedics NeuroScan, USA) for data pre-processing and extraction of ‘decision’ event related responses. The data were inspected by eye for artifacts and bad channels were rejected. Offline re-referencing, using average reference was then performed [35].

Filtering and artifact rejection criteria were set as in experiment 1. We corrected for eye-blinks (and other artifacts) segment-wise by rejecting data trials where the maximum exceeded 100 µV in any of the EEG or EOG channels. Moreover, because of the dynamic stimuli used in this experiment, we used independent component analysis (ICA) for signal “correction”, in particular saccade potentials attenuation, based on all electrodes (including 4 EOG channels) [24]. We identified the ocular component based on the scalp topography (higher activity around the orbits) and its relation to EOG channel peaks, as described by Keren et al, 2010 [24].

Data were segmented into epochs (−1500 ms to 500 ms) locked to the response or to the middle of the trial, for the perceived trials (perceptual “discovery” moment) and non perceived trials respectively. Epochs were separated accordingly to the response (perceived, no-perceived). To guarantee that the baseline was equal between different trials, only trials with responses between 1 s to 11 s were considered for analysis.

Topography, time-frequency, phase synchrony and additional statistical analysis were implemented in Matlab®(v.R2010a, The MathWorks, USA).

### Time-frequency and phase synchrony analysis

Time-frequency analysis was performed as in Uhlhaas, et al. (2006) [35], [36] and is also described elsewhere [17], [35], [36], [48]. Signals were time-frequency-analyzed using the pseudo Wigner-Ville transformation. For every time window and frequency bin (frequency resolution of 1 Hz/frequency bin) the amplitude and phase were computed, using Matlab®, in the high-beta/gamma frequency range (15 to 90 Hz in steps of 1 Hz) and in the time period of the epochs described above with EOG correction performed as described in Keren, et al. (2010) [24]. From these phase values we calculated the phase-locking value (PLV), that measures the inter-trial variability of the phase difference [35]. PLV looks for latencies at which the phase difference between the signals varies little across trials at the target frequency. Because we were interested in long-range coordination of neural activity, for a given time window, the phase difference was calculated between all electrode pairs, and the stability of phase difference evaluated through all the trials across a large frequency range [17], [35], [36]. Coherency is an indicator of neural synchrony but, this phase calculation can be affected by volume conduction since activity of a single source is measurable in many electrodes. To avoid spurious synchrony, we computed PLV between sources, using the imaginary component part of coherence (ImCoh) as a measure for functional connectivity at the sensor level [37], [49]. As described by Nolte et al 2004 [37], ImCoh more directly reflects true interaction. We performed this analysis as it is implemented in Source Information Flow toolbox for EEGLAB (SIFT version 0.9.7-alpha) [50], [51]. To further examine the ImCoh we plotted the head-in-head plots to visualize interactions between conditions over all channels (pairs of channels) [49], [52]. The topographies are plotted at each electrode position and represent the connectivity strength (ImCoh) between that given channel and all other channels for each frequency band (15–30 Hz, 30–45 Hz, 45–60 Hz, 60–75 Hz).

Time-frequency analysis was performed as in Uhlhaas, et al. (2006) [35], [36] and is also described elsewhere [17], [35], [36], [48]. Signals were time-frequency-analyzed using the pseudo Wigner-Ville transformation. For every time window and frequency bin (frequency resolution of 1 Hz/frequency bin) the amplitude and phase were computed, using Matlab®, in the high-beta/gamma frequency range (15 to 90 Hz in steps of 1 Hz) and in the time period of the epochs described above with EOG correction performed as described in Keren, et al. (2010) [24]. From these phase values we calculated the phase-locking value (PLV), that measures the inter-trial variability of the phase difference [35]. PLV looks for latencies at which the phase difference between the signals varies little across trials at the target frequency. Because we were interested in long-range coordination of neural activity, for a given time window, the phase difference was calculated between all electrode pairs, and the stability of phase difference evaluated through all the trials across a large frequency range [17], [35], [36]. Coherency is an indicator of neural synchrony but, this phase calculation can be affected by volume conduction since activity of a single source is measurable in many electrodes. To avoid spurious synchrony, we computed PLV between sources, using the imaginary component part of coherence (ImCoh) as a measure for functional connectivity at the sensor level [37], [49]. As described by Nolte et al 2004 [37], ImCoh more directly reflects true interaction. We performed this analysis as it is implemented in Source Information Flow toolbox for EEGLAB (SIFT version 0.9.7-alpha) [50], [51]. To further examine the ImCoh we plotted the head-in-head plots to visualize interactions between conditions over all channels (pairs of channels) [49], [52]. The topographies are plotted at each electrode position and represent the connectivity strength (ImCoh) between that given channel and all other channels for each frequency band (15–30 Hz, 30–45 Hz, 45–60 Hz, 60–75 Hz).

Time-frequency (induced) and phase were then normalized to the baseline. The normalization involves subtracting the baseline average and dividing by the baseline standard deviation on a frequency by frequency basis [35], [36]. Baseline was chosen accordingly to the task. In the first experiment the baseline was set to pre-stimulus interval (avoiding inclusion of post-stimulus oscillations in the baseline segment). For the second experiment, using dynamic stimuli, baseline time-window spanned the no-perception time window (the time before perceptual decision). After normalization, individual time-frequency and phase synchrony data were averaged through subjects to obtain grand-averages.

### Statistical analysis

The alpha level was set at 0.05 for all tests. After verifying for normality (Kolmogorov-Smirnov), statistical tests were performed. After a visual inspection of the results we focus and tested four frequency bands of 15 Hz length each (15–30 Hz, 30–45 Hz, 45–60 Hz, 60–75 Hz) to assess the significance of the oscillatory patterns with paired t-test and Wilcoxon signed rank test. The Bonferroni-Holms correction for multiple comparisons at level alpha was applied when appropriate [53].

As a further statistical analysis we performed SVM classification applied to matched perceived/unperceived time window. We have performed classification of perceptual “discovery” responses of experiment 2 for the subjects (n = 6) that had at least 10 non-perceived trials. In 8 subjects the number of unperceived trials was too low for classification. However, single subject statistics were highly consistent across all 6 eligible subjects’ permiting proof of concept validation. We were conservative in subject selection for SVM but we guaranteed that we had enough number of trials for both states. The algorithm classified between perceived/unperceived trials. We used linear SVM implemented in the libSVM library [38] based on time-frequency data. Data were divided into training and test sets. The classifier used 3 seconds at the end of the movies (for the perceived movies only trials with responses not overlapping this period were considered; in this way we guaranteed non contamination of overlaid motor responses). It used time-frequency data features based on the average over time in occipito-parietal channels (C1/Z/2, CP1/3/Z/2/4, P1/Z/4, PO9/7/5/3/Z/4/6/8/10, O1/Z/2) for each averaged time-point per frequency band. To determine the best regularization parameter, we used a 3-fold cross-validation scheme. Thirty repetitions were computed with random distribution of data among folds. We used a permutation test approach [54], [55] to evaluate the statistical significance of the classification's results. In this procedure, labels are assigned randomly to the example trials, then the classifier is trained on the task with the permuted condition labels and finally the generalization performance is tested with a “leave-one-out” cross-validation strategy.

## Results

### Behavioural data

Hit rates for Mooney tasks were as follows, task 1: mean 89.75±8.46% for faces and 90.36±5.96% for guitar stimuli; task 2: mean 91.87±7.28%. As an additional measure of reliable perception we computed the overall group % of wrong categorization. 9.22% of stimuli were incorrectly categorized as faces or guitars.

In task 2 (Mooney dynamic stimuli) participants required a mean detection time of 4.30±2.95 s. When converting time into an angular rotation required for detection, a mean value of 86° from the inverted position was obtained, suggesting that the object had to be at least close to the orthogonal position for recognition. These results are consistent with the face inversion effect [27], [56].

### Neurophysiological Results

#### Emergent perception of faces elicited by both short lived or dynamic ambiguous two-tone (Mooney) stimuli is related to increases in gamma-band activity in visual posterior brain regions

**Figure 2:**
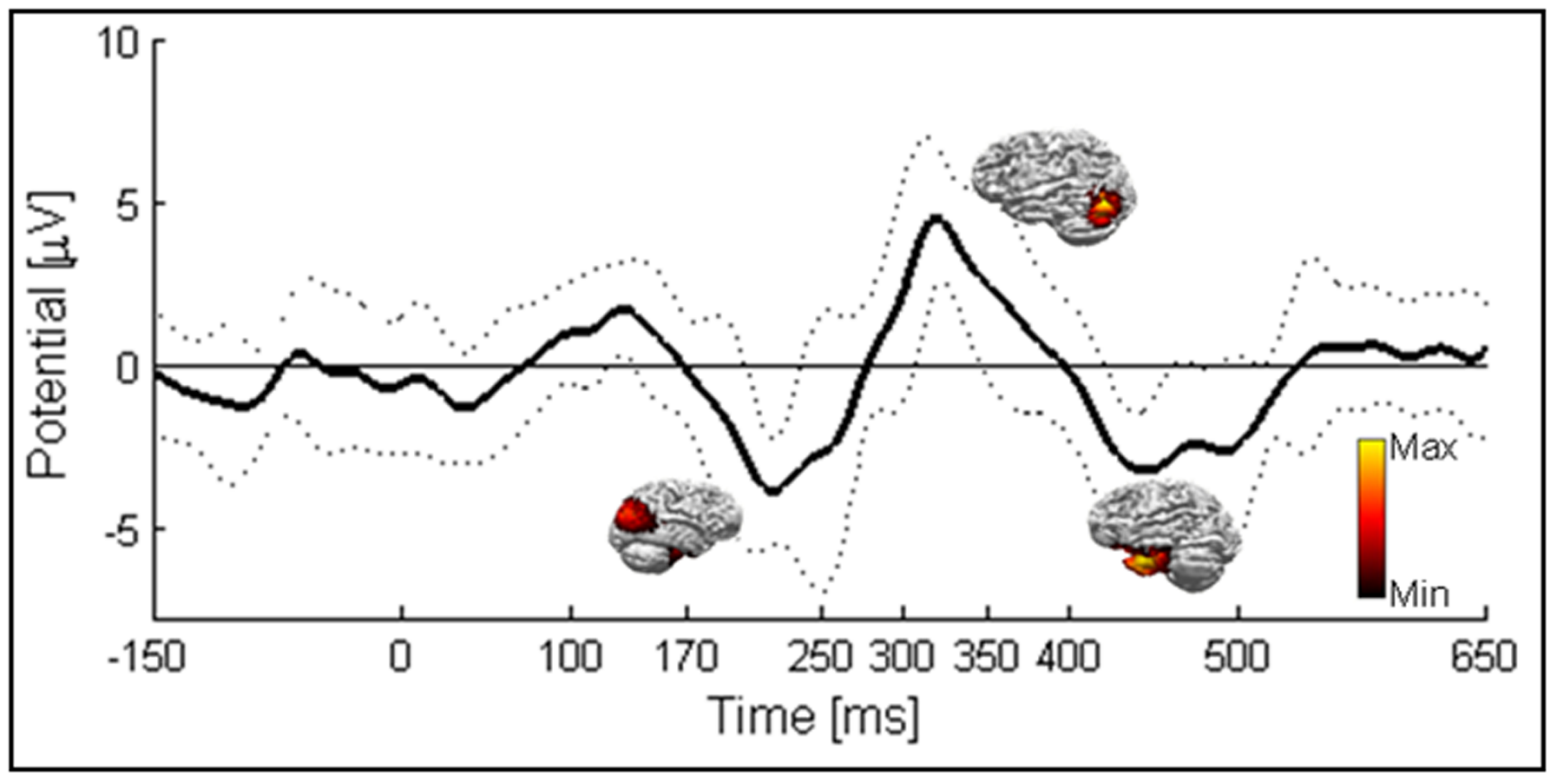
Group average ERP for experiment 1 - Mooney Rapid Visual Presentation. Baseline is set to −250 ms to 0 ms. ERP peaks negatively around 220 ms and is consistent across subjects (line points means standard deviation). Source distribution maps show sLORETA standardized current density for the three different peaks (hot colors signals the highest current density reconstruction values).

An ERP that peaks negative at 220 ms after face/guitar onset was found conspicuously for the short-lived stimuli in experiment 1 (see Figure 2). This peak preceded a positive peak at 300 ms that is a known mark of target rare events [57]. We performed source localization that revealed increased activity from occipito-parietal regions for the N220 component and more inferior temporal sources of activation for the P300 to the N400 components both for Mooney faces or guitars (see source mapping in Figure 2).

**Figure 3:**
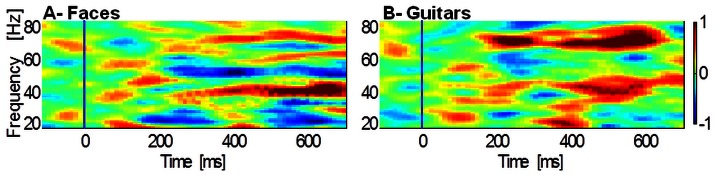
Time-frequency plot for the posterior channels– Experiment 1. Data are locked to the onset of the oddball salient frame (A: Face; B: Guitar), baseline corrected for the pre-stimulus interval and normalized for the baseline interval. Significant differences revealed prominent gamma activity for face trials at low frequency (30–45 Hz, 373–685 ms; z = 4.14; p corrected<0.001) as well as for guitar trials both for low (30–45 Hz, 370–600 ms; z = 5.06; pcorrected<0.0001) and high frequencies (60–75 Hz; 166–641 ms; z = 10.33; pcorrected<0.0001) when comparing with the baseline. Blue line marks the beginning of the target stimuli; Color scale: normalized units.

Regarding time-frequency analysis we found increased gamma activity specifically related to the perception moments amongst noise pictures in experiment 1 (Figure 3). These patterns were indeed phase-locked to the detection of the target faces or objects and appeared in both low and high gamma frequency bands (30–45 Hz and 60–75 Hz respectively). In order to confirm these findings we performed statistical comparisons between target and frequent conditions with Wilcoxon rank-sum tests for each time point and frequency band. Significant differences revealed gamma activity was increased for face trials at low frequency (30–45 Hz, 373–685 ms; z = 4.14; p corrected<0.001) as well as for guitar trials both for low (30–45 Hz, 370–600 ms; z = 5.06; pcorrected <0.0001) and high frequencies (60–75 Hz; 166–641 ms; z = 10.33; pcorrected<0.0001).

**Figure 4:**
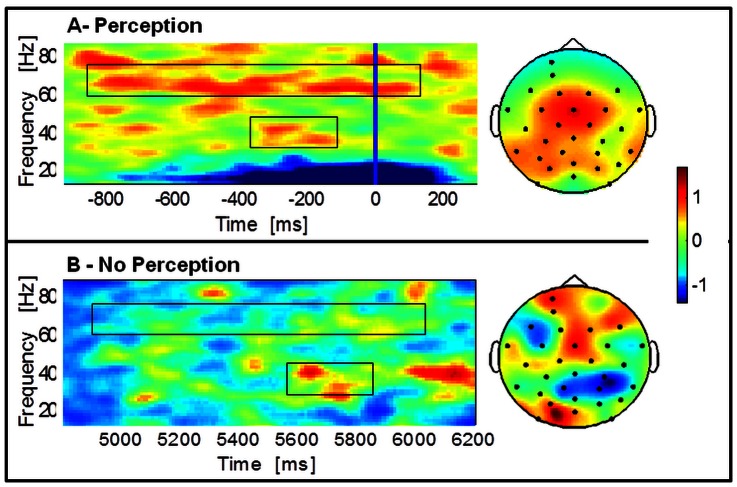
Time-frequency representations and topographies in the perception (A) and no perception (B) conditions– Experiment 2. A: group average responses to Mooney dynamic stimuli locked to response onset (blue line), baseline corrected and normalized for the baseline interval (fixation and stimulus); B: group average activity for the non- perceived trials. Topography plots for high and low gamma response are shown for perception and no perception conditions respectively. The topographies are averaged across the time window (−800 – 0 ms) for the higher gamma-band (60–75 Hz) and lower gamma-band (30–45 Hz; top right and bottom right, respectively). The gamma-band signal is expressed as relative power change during perception compared to baseline, averaged across all channels. Note that the frequency band where the effect size is highest is the higher gamma-band. Boxes highlight low (30–45 Hz) and high (60–75 Hz) gamma bands, here and in subsequent figures.

In experiment 2, we replicated these findings. We analyzed temporal patterns of activity prior and after the perceptual identification of faces using dynamic Mooney stimuli. Our difficult stimulus conditions whereby stimuli start from an inverted position often lead to a delayed recognition moment, as expressed by the angle of stimulus rotation that is present at the moment of perceptual report (see behavioural results). We have found increases in gamma activity that starts prior to the perceptual report (see time-frequency plots in Figure 4A and 4B for perception and no perception results respectively). We found gamma-band peaks of activity patterns at two frequencies (group averaged gamma peaks: 32.69±12.59 Hz and 70.33±7.75 Hz) in response to moments of perception. In Figure 4, for each time-frequency plot, we depict the topography maps of the gamma response. We found increased central activity for the perception condition (60–75 Hz gamma response) and a decrease over the parietal regions for the no perception condition (30–45 Hz gamma response).

Comparison of no perception *vs.* perception during a time interval corresponding to the second before perceptual report revealed significantly higher gamma-band activity for Perception in the frequency bands 30–45 Hz, 45–60 Hz and 60–75 Hz (see Table 1 for statistics).

**Table 1:** No perception*vs.* Perception statistics for experiment 2.

Frequency range (Hz)	p	t	
15–30	0.011187	−2.7584	n.s.
30–45	6.7571E-06	−5.7881	**Perception** higher than no perception
45–60	4.3518E-07	−6.9535	**Perception** higher than no perception
60–75	4.7501E-14	−16.1611	**Perception** higher than no perception

Results of statistical t-tests when comparing perception and no perception during the second before button press. We tested four frequency bands. p and t values are shown and differences were considered significant for p<0.0025 (corrected for multiple comparisons). Perception show increased activity for higher gamma frequencies.

We did also observe a peri-stimulus response reduction in the lower frequency (15–30 Hz) beta band. This band in time-frequency plots was only observed when a motor response was required.

#### Frontal gamma-band activity is decision related

**Figure 5:**
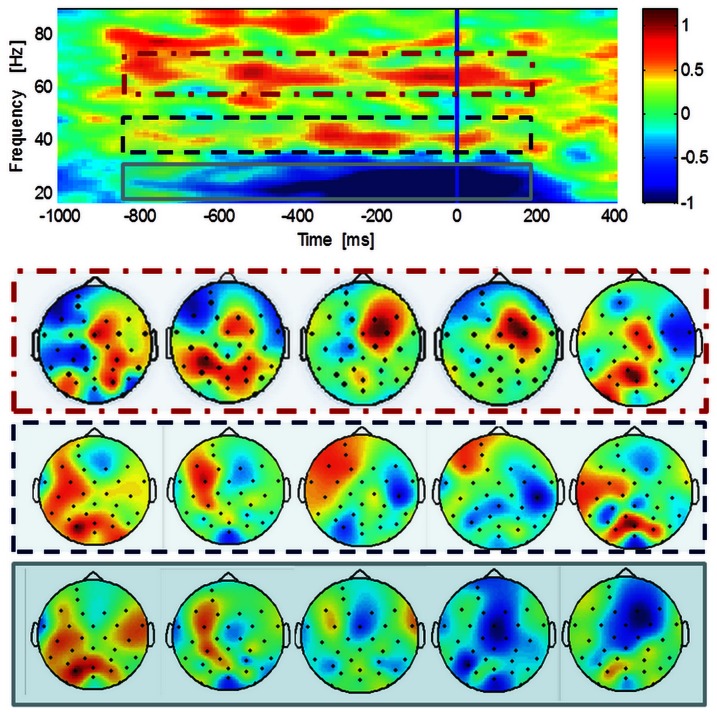
Normalized time-frequency plots in 2D scalp maps (experiment 2). These maps are plotted for the channels marked as a black point in the 2D topographies in five consecutive time windows of 200 ms. The red-dashed row is associated with higher gamma-frequencies: activity is increased not only in occipital electrodes but seems to change its ‘centre of gravity’ during the time to more parieto-frontal areas. The blue-dashed row shows topographic maps for low-gamma. Gray row show the deactivation at the lowest frequencies (beta) that seems to have their source in central regions usually reported as motor areas.

We observed significant gamma-band activity in frontal electrodes subsequent to occipital gamma related activation (see Figure 5) suggesting the occurrence of these temporal patterns in anterior locations until the moment of perceptual decision.

Accordingly, for the higher frequencies, activity is increased not only in occipital electrodes but seems to change its ‘centre of gravity’ during the time to more parieto-frontal areas. The deactivation at lower (beta) frequencies seems to have its source in central sensorimotor related regions.

#### Phase synchrony increases before the recognition moment

**Figure 6:**
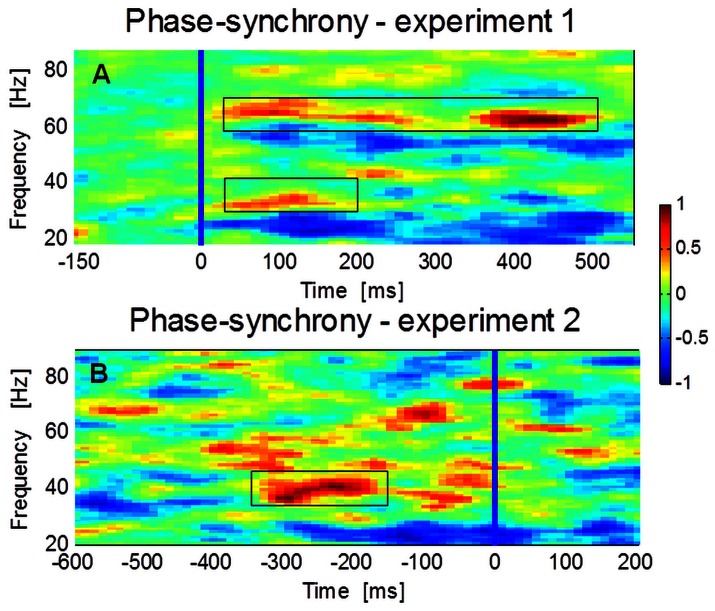
Phase synchrony results for the two experiments. A: Phase synchrony for task 1, synchrony is locked to the “target” face. Boxes highlight the higher synchronization at both low (30–45 Hz; 200–373 ms; z = 4.69, pcorrected<0.0001) and high (60–75 Hz; 178–629 ms; z = 5.46, pcorrected<0.001) gamma frequency bands (Figure 6A). B: Increased synchronization for the lower gamma-band (30–45 Hz) appears during task 2 (−324- −166 ms; z = 3.0516, pcorrected<0.003) before the perceptual report (blue line).

**Figure 7:**
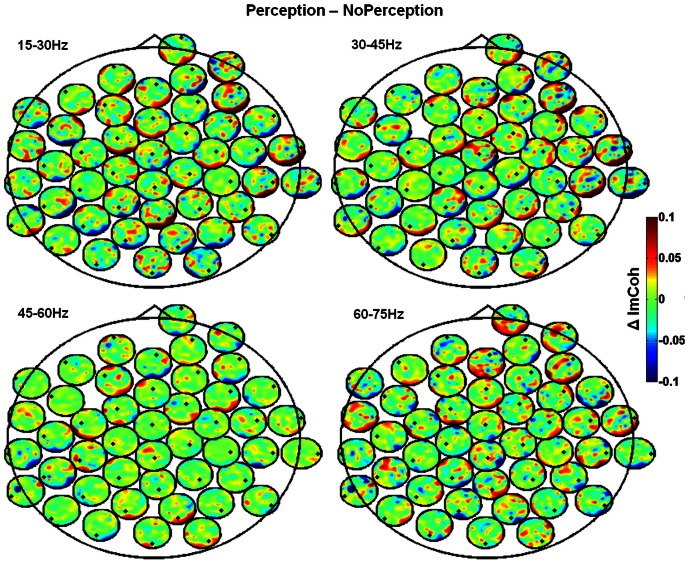
Head-in-head plots for the imaginary part of coherency at each frequency band. ImCoh is represented between all channel pairs time-averaged for the second before the button press. The difference between perception and no perception conditions is shown. Each small black dot corresponds to the position of the reference electrode in terms of connectivity. Note the link between occipital and frontal sites in the gamma range. Colorbar codes connectivity.

After normalizing to periods corresponding to the no perception states, group averages showed a burst of synchronization that appears 400 ms prior to detection. In experiment 1 (suddenly recognizable objects) we observed higher synchronization at both low (30–45 Hz; 200–373 ms; z = 4.69, pcorrected<0.0001) and high (60–75 Hz; 178–629 ms; z = 5.46, pcorrected<0.001) gamma frequency bands (Figure 6A). In experiment 2 increased synchronization (30–45 Hz) indexes were detected just prior (−324- −166 ms; z = 3.0516, pcorrected<0.003) to the perceptual report (Figure 6B). We have observed a consistent pattern of increased synchronization in the both experiments for the lower gamma frequency band. Moreover we have analyzed the imaginary component part of coherence (ImCoh) as a measure of synchrony that controls for volume conduction spurious activity and we found different connectivity patterns for the different conditions suggesting that distinct neurophysiological mechanisms were involved associated with the upcoming object recognition. Figure S1 shows ImCoh plots as function of frequency. We found increased connectivity for the same frequency bands and time-intervals thus replicating the results of phase-synchrony. Figure 7 shows the ImCoh head-in-head plots difference between perception and no perception conditions. The connectivity is stronger for the topographic distributions that contain frontal and right occipito-parietal regions although one cannot make inferences about the directionality of information flow [49].

#### SVM data driven analysis shows that temporal patterns of gamma activity are informative in the classification of perception vs. no perception states

The SVM classifier was able to classify without an apriori hypothesis the perceived/non-perceived trials of the Mooney dynamic task with high accuracy (>95%), above chance for all subjects where a significant number of no perception trials were available. A group average result for accuracy, sensitivity, specificity and balanced accuracy is shown for each frequency band in Table 2. The classifier performed with balanced accuracy above 95% for all these bands (that are matching the time-frequency results). The permutation results yielded p-values bellow 0.001 in all of the cases, for the classification of perceptual states (which is remarkable even if the set of subjects with a sufficient number of trials for classification was low). The likelihood that this would happen by chance even at a group level is very low. The contribution of each sub-band to the classification was based in an increase in perception related activity.

**Table 2:** SVM classification results.

LibSVM: No Perception *vs.* Perception				
Frequency Bands (Hz)	Accuracy	Sensitivity	Specificity	Balanced Accuracy
15–30	98.04±1.29	99.07±0.93	91.24±6.59	95.15±4.00
30–45	98.11±1.31	99.34±1.06	91.78±5.25	95.56±2.96
45–60	97.87±1.43	98.67±0.99	92.93±6.52	95.80±3.91
60–75	97.83±1.51	98.96±0.94	93.34±6.88	96.15±4.20

Frequency data from occipito-parietal electrodes were used as classification features to separate between perceptual states. Only subjects with >10 non perceived trials were used. A group average of accuracy, sensitivity, specificity and balanced accuracy is shown for each frequency band. These bands are matching the time-frequency results (Table 1). We performed a permutation test for each subject and all p values were below threshold (p<0.001). Classification was successful for all tested subjects.

## Discussion

This study provides a direct link between gamma-band temporal patterns and the presence versus absence of emerging holistic perception of variable onset. We investigated visual perceptual recognition moments based on EEG/ERP analysis with two different experiments. The novelty of our study lies in the fact that we departed from classical paradigms which are based on contrasts between stimuli conditions that are fixed in time. Our approach improves conventional designs by using short lived rapid visual presentation of many events with 1/30 likelihood of target presence or novel dynamic approaches whereby percepts are variable in which concerns the moment of recognition. In the latter case, this ensured that the moment of perception of an emergent global pattern was variable. This way we could directly compare perception *vs.* no perception states for the same stimuli.

In the first task, the short lived presentations yielded a characteristic ERP with a negative peak at 220 ms. Previous studies have shown a similar component for decision related paradigms [58]. The emergence of this negative peak is very common and can be related with the decision making demands of our paradigm as reported previously by others [59] even in other sensory modalities [57]. Moreover, our ERP data shows a clear P300 peak that appears in response to the rare target stimuli. In this experiment, we have found that increased gamma-band patterns appear in response to brief moments of object percepts.

This result was replicated in the second experiment, using dynamic stimuli. The topography plots for the perception condition at each frequency band show a broad distribution of the activity over the scalp. In this case, higher gamma-band activity appears in more anterior areas, possibly corresponding to decision related central regions. Interestingly, for the lower band of no perception states activity appears reduced over the occipito-parietal areas.

Both high beta/low gamma (30–45 Hz) and high gamma (60–75 Hz) frequencies showed higher synchrony but with an expected decrease in amplitude for the lower band, for perceived objects (faces or guitars, which are both very prototypical objects). Although the brief presentation paradigm show good evidence for a perceptual role of distinct gamma-band patterns in the emergence of percepts, our dynamic paradigm extended this notion by taking advantage of the well known role of face inversion in holistic processing [27], [30], [32], [33], [60]. The face-inversion effect, has been replicated by behavioral studies [61] but has also been reported by other brain imaging studies (e.g. [56], [62]). Our manipulation uses the bias of holistic processing in Mooney stimuli. It delayed the time of perceptual discovery from stimulus onset through a gradual rotation from inverted to upright position because objects are mostly perceived far from the inverted position. This way, sensory processing was separated from perception. We would like to emphasize (as reported in Rebola et al., 2012 [29]) that one departs from a configuration not favoring holistic perception to one favoring an holistic perceptual interpretation, as also discussed by Jemel and colleagues 2009 [27]. By delaying the moment of global integration or rendering it unpredictable this paradigm focused on global gestalt mechanisms instead of local sensory to noise levels. The moment a coherent visual stimulus is perceived was therefore variable in its time of occurrence as well as in the Rapid Visual Presentation paradigm. Nevertheless, as reported by Melloni et al., 2011 [63] the expectation for the stimulus at a short time scales may affect peak signal latency and amplitudes.

The analysis of phase synchrony patterns showed that object perception was associated with a burst of synchronous activity in low frequency gamma-band components in both tasks. This synchrony pattern for the lower frequency band (most specific in task 2 but also present in task 1) irrespective of amplitude of gamma-band changes suggests that, at lower gamma-band frequencies, a different neurophysiological process [35] associated with the upcoming object recognition, was involved. Interareal synchrony between areas has been reported as a mechanism for binding of information across different brain regions [11]. To validate this increased interaction we looked to the ImCoh as a reliable measure for neuronal interactions that is insensitive to volume conduction artifacts [37], [49] and we found a similar pattern of activity with increased connectivity for the same frequency bands and time-intervals (see Figure S1). It is accepted that ImCoh represents brain connectivity at the sensor level [37], [52], [64]. According to this view, the connectivity patterns we found can be related with the interaction between dorso-ventral stream regions involved in object perception and anterior areas usually reported as decision related. Remarkably, we found a decrease in gamma amplitude during an increase in synchrony. These patterns are evidence that the synchrony increase was not caused spuriously by neither a change in power of a common source or a volume conduction artifact.

These results shed light on the mechanisms underlying perceptual object processing and decision making and provide support for the role of gamma-band frequency patterning and synchrony in the well known binding problem [8], [9], [65]. Our data supports a functional role for distributed spatiotemporal patterns of gamma-band activity and synchronization in perceptual decision. Together, these findings provide support for the claim that gamma-band activity is a signature of emergent holistic perceptual states.

An additional contribution of this study was the independent validation by data driven (non-hypothesis constrained) approaches. Support vector machine classification approach enabled us to directly distinguish between non perceptual *vs.* perceptual states, based on time-frequency features in the gamma frequency band. This SVM approach is a proof of concept that worked in all subjects that were eligible for classification in terms of number of trials. Classification balanced accuracy, sensitivity and specificity were higher than 95% thereby emphasizing perception related neurophysiological signatures. This analysis should be viewed in light of our evidence that sustained activity was dominant in the gamma-band, particularly in perception states [66]. These results show that a broad range of frequencies is informative, corroborating the tenet that the brain uses different oscillatory bands to code different information [17], [39], [41]. This statistical classification of perceptual states using an SVM approach suggests an important functional role for gamma activity patterns that can be generalized to ambiguous percepts. This analysis provides evidence that time-frequency patterns at gamma-band frequencies provide sufficient information to infer about perceptual states in a data driven manner.

In sum we have found evidence that gamma-band features can differentiate perceptual versus non perceptual states, as confirmed by SVM classification. We conclude that a functional role for distinct distributed spatiotemporal patterns of gamma-band activity can be identified for the moment a holistic object percept is formed.

## Supporting Information

Figure S1 **Representation of imaginary coherence (ImCoh) over all channels (pairs of channels) as function of frequency.** For experiment 1 (top panel) baseline was set to the interval before stimulus presentation. For experiment 2 (bottom panel) a baseline was subtracted consisting of the coherence time-averaged in the interval −1200 - −1000 ms. Colorbar codes imaginary coherence. Increased connectivity for the high beta/low gamma and high gamma band is present thus replicating the results of phase-synchrony.
(TIF)
