# Supplementary figures and images for: To Perceive or Not Perceive: The Role of Gamma-band Activity in Signaling Object Percepts
Source: PLoS One. 2013 Jun 13;8(6):e66363. doi: 10.1371/journal.pone.0066363 (PMC3681966; doi:10.1371/journal.pone.0066363)

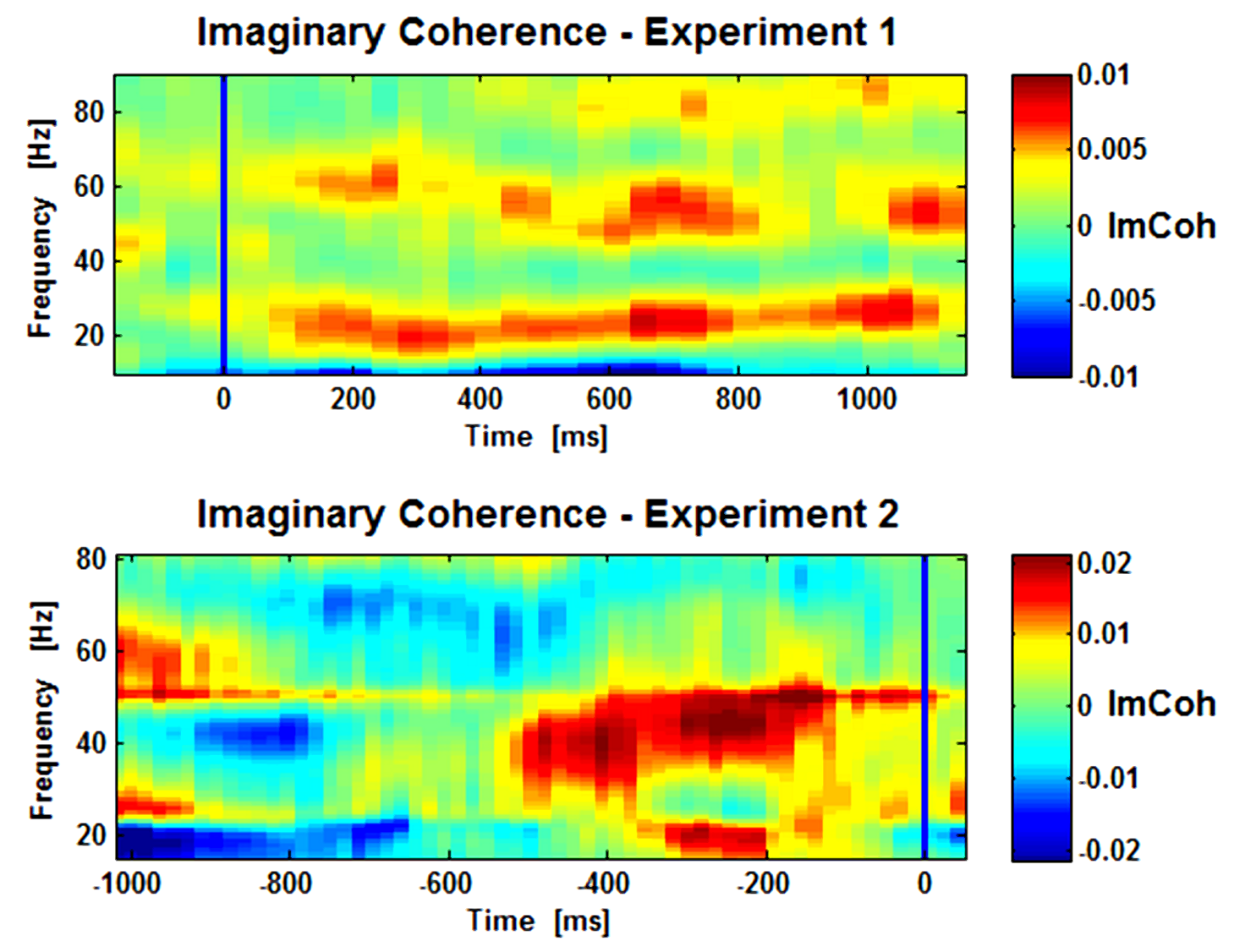

Supplement: Figure S1 — Representation of imaginary coherence (ImCoh) over all channels (pairs of channels) as function of frequency. For experiment 1 (top panel) baseline was set to the interval before stimulus presentation. For experiment 2 (bottom panel) a baseline was subtracted consisting of the coherence time-averaged in the interval −1200 - −1000 ms. Colorbar codes imaginary coherence. Increased connectivity for the high beta/low gamma and high gamma band is present thus replicating the results of phase-synchrony. (TIF) [file pone.0066363.s001.tif]
